# The tricuspid valve in hypoplastic left heart syndrome: Echocardiography provides insight into anatomy and function

**DOI:** 10.3389/fped.2023.1145161

**Published:** 2023-03-27

**Authors:** Tara Bharucha, Nicola Viola

**Affiliations:** ^1^ Department of Paediatric Cardiology, University Hospital Southampton NHS Foundation Trust, Southampton, United Kingdom; ^2^ Department of Congenital Cardiac Surgery, University Hospital Southampton NHS Foundation Trust, Southampton, United Kingdom

**Keywords:** hypoplastic left heart syndrome, tricuspid valve, echocardiography, congenital heart disease, ventricular function

## Abstract

Tricuspid regurgitation (TR) is commonly seen in surgically palliated patients with hypoplastic left heart syndrome, and when significant, is associated with an increase in both morbidity and mortality. Tricuspid valve dysfunction appears to be the result of a combination of inherent structural malformations and the unique physiological circumstances resulting from right ventricular pressure and volume overload. Valve dysfunction evolves rapidly, and manifests early on in the surgical pathway. Whilst traditional echocardiographic imaging can identify anatomical defects and dysfunction resulting in varying degrees of regurgitation even at early stages, more sophisticated investigations such as 3D echocardiography, strain imaging and transesophageal 3DE might prove useful to better demonstrate the complex interactions between abnormal anatomy of the valve complex, ventricular function, mechanical synchrony, and TR. Recognition of specific mechanisms of TR can enhance patient-specific care by directing precise surgical interventions and by informing the best timing for intervention on the valve.

## Introduction

Hypoplastic left heart syndrome (HLHS) represents a spectrum of congenital malformation of the left heart and left-sided vascular structures (1, 2). A series of surgical interventions are offered in order to create a functionally univentricular circulation, in which the right ventricle (RV) functions as the systemic ventricle (3, 4).

The tricuspid valve (TV) therefore must perform in the systemic circulation, and experiences haemodynamic conditions that are quite different to its circumstances in a normal, biventricular circulation.

Tricuspid regurgitation (TR) is commonly seen in surgically palliated patients. TR of at least moderate degree is associated with poorer outcomes for patients with functionally univentricular circulations, with an increase in both morbidity and mortality (5–8). Hence, an understanding of the mechanisms of TR could be important in clinical management, and in striving to improve patient outcomes and survival. One possible mechanism of the occurrence of TR is that it is a result of anatomical abnormalities, and another is that it is a function of the unique physiological circumstances in which the TV is placed.

The mechanisms of dysfunction of the tricuspid valve are often difficult to fully elucidate using traditional imaging, and also evolve over relatively short periods of time, including in the interval between palliative surgical stages. Additionally, there are complex interactions between ventricular function, mechanical synchrony, and TR.

The multifactorial nature of the aetiology of TR makes it difficult to tease out specific mechanisms in individual patients. Effective surgical intervention can improve patient outcomes (8), but relies on pre-operative recognition and clarification of possible surgical targets.

## Tricuspid valve anatomy

**Figure 1:**
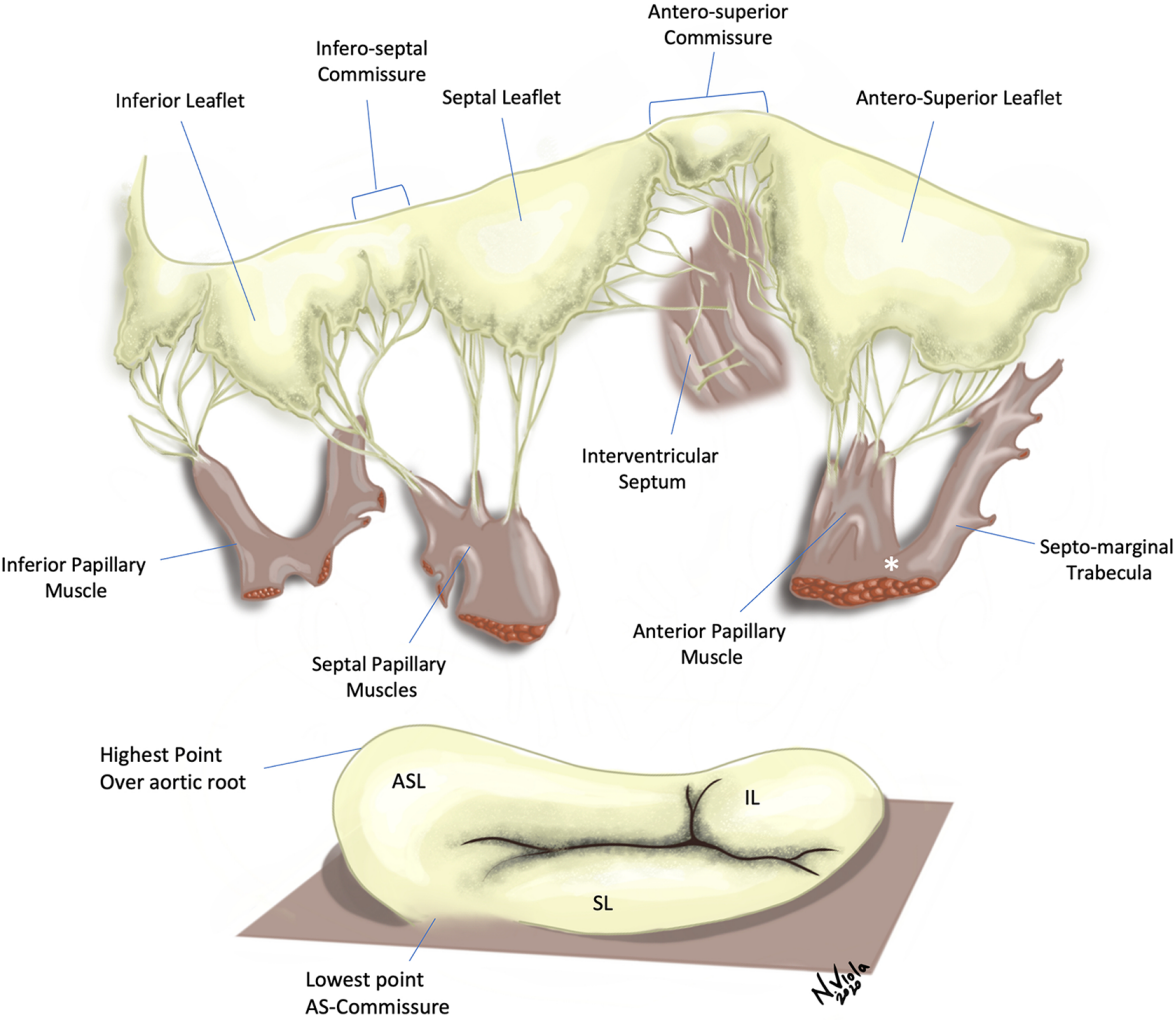
Tricuspid valve with leaflets and subvalvar apparatus. Top Panel—Several chordal attachments to the interventricular septum can be seen, both directly or through small septal papillary muscles. The anterior papillary muscle is connected to the septo-marginal trabecula *via* the moderator band (*). Bottom Panel—The tricuspid valve annulus has a wave-like, saddle-shaped form. Note the lack of discernible annulus in proximity of the antero-septal commissure. ASL, Antero-superior leaflet; SL, Septal leaflet; IL, Inferior leaflet.

A normal tricuspid valve is composed of four components, namely the leaflets, the papillary muscles, the chordal apparatus and the valve annulus (9) (Figure 1).

Usually there are three leaflets, described in an attitudinally appropriate manner (relative to the anatomic position in the body) as anterior-superior, septal and inferior, with the anterior-superior being the largest. Papillary muscle arrangement is highly variable; generally, there are two main muscles, the anterior and the posterior. The third, the septal papillary muscle, displays the most inconsistency, being often absent, or alternatively, multiple. The chordae attach to the papillary muscles, and together these form the tensor apparatus for the TV. The normal tricuspid annulus is D shaped, and is a dynamic structure, changing shape considerably during the cardiac cycle. It is mainly fibrous, but contains muscle bands, especially in the anterosuperior portion, and is less distinct than the mitral annulus.

In hearts with HLHS, the TV anatomy is extremely variable. A study of 82 pathological specimens of HLHS (10) showed that there are differences in the TV in HLHS compared to normal hearts, but also differences within the anatomical subtypes of HLHS. Variation in the number of leaflets was common, with 12% of patients having a bicuspid TV, and quadricuspid TVs being occasionally seen. One third of the specimens showed moderate or severe dysplasia of the leaflets, including thickening, reduced mobility or nodular malformations. Accessory orifices were also noted in a small number of cases. Papillary muscle variability was common, and the septal papillary muscle was frequently absent in patients with a patent mitral valve; these patients have very abnormal septal geometry, which is likely to be a factor in this finding.

It is valuable for both the echocardiographer and the surgeon to be aware of these anatomical differences and variability, enabling surgical planning and also aiding in prognostication.

## Surgical intervention

Surgical intervention to the TV can reduce TR severity and may improve functional outcomes of patients with HLHS (8, 11, 12). However, TV repair improves survival in only a subset of patients (13), and there is a risk of necessity of late reintervention on the TV (12) which suggests that intervention specifically targeted to the individual patient's abnormality or constellation of abnormalities is desirable. Honjo and colleagues (13) report lower survival for a mixed group (more than half with HLHS) of patients with single ventricle malformations who underwent atrioventricular valve repair, and they note that ventricular dysfunction and dilatation was associated with repair failure and death. However, patients who attained a successful repair (one that resulted in less than moderate regurgitation) with preserved ventricular function achieved survival equivalent to case-matched controls who did not undergo valve repair.

Surgical inspection of the TV does not facilitate appreciation of the contribution of dynamic changes in the annulus, nor of motion abnormalities of the valve (14). It can however indicate or confirm the presence of leaflet tethering, which seems to be a significant risk factor for suboptimal repair (15). Repair is widely performed and often necessary but established surgical approaches, such as obliteration of the posterior leaflet (PLO), though effective in some, may not address the specific mechanisms of dysfunction in each individual patient (11). For example, TR arising in the anteroseptal commissure is poorly corrected by this technique, which also does not address prolapse or tethering of leaflets. Annuloplasty is widely adopted as an effective technique as part of surgical repair, but often several other techniques are employed in isolation or combined, such as direct closure of fenestrations, papillary muscle repositioning, multiple or selected commissuroplasties (16). Timing of repair is also an important contributing factor to repair outcomes overall: early onset of anteroseptal prolapse or tethering, before stage II, is associated with poorer outcomes, with surgical interventions aimed at partial annular instability or chordal insufficiency in infancy being less effective (17).

## 2D echocardiography

Echocardiography can demonstrate TV structural abnormalities that may be amenable to surgical techniques, and also, notably, is able to demonstrate the valve in motion and therefore reveal the functional importance of valve pathology (18). The functional performance of the TV can change rapidly in the early stages of the surgical pathway. Approximately one third of infants display TR of at least mild grade on echocardiography prior to stage II intervention (5, 19). Echocardiography can accurately identify some anatomical causes of TR, with anteroseptal commissure prolapse and leaflet tethering commonly recognised as important features and also as risk factors for residual TR following surgical repair (17).

An echocardiographic study (14) sought to compare echocardiographic findings of valve abnormalities to surgical inspection of the TV at the time of TV repair in patients with HLHS, often performed at the time of the second or third surgical stage of palliation.

**Figure 2:**
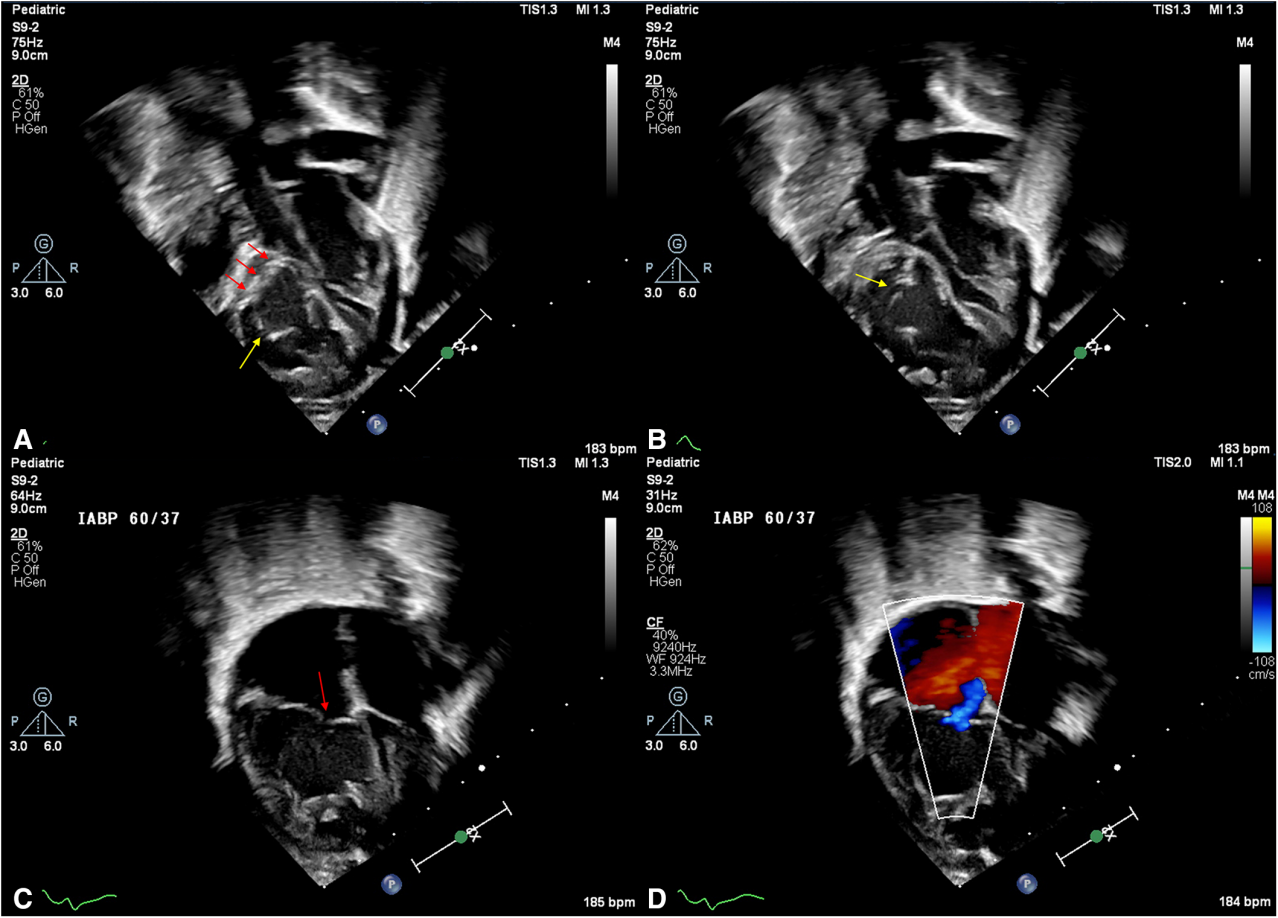
Tricuspid valve malformations in HLHS. (**A**) Subcostal view of the tricuspid valve in early-mid diastole. The anterior leaflet free margin is bright and thickened (red arrows), and a deep cleft is noted at the antero-inferior commissure. (**B**) Subcostal view in late diastole. An additional cleft and nodules are noted on the anterior leaflet (yellow arrow). (**C**) Apical 4 chamber view in early systole. Note the poor coaptation between anterior and septal leaflets (red arrow) and, (**D**) colour Doppler interrogation in the same view showing mild tricuspid regurgitation.

**Figure 3:**
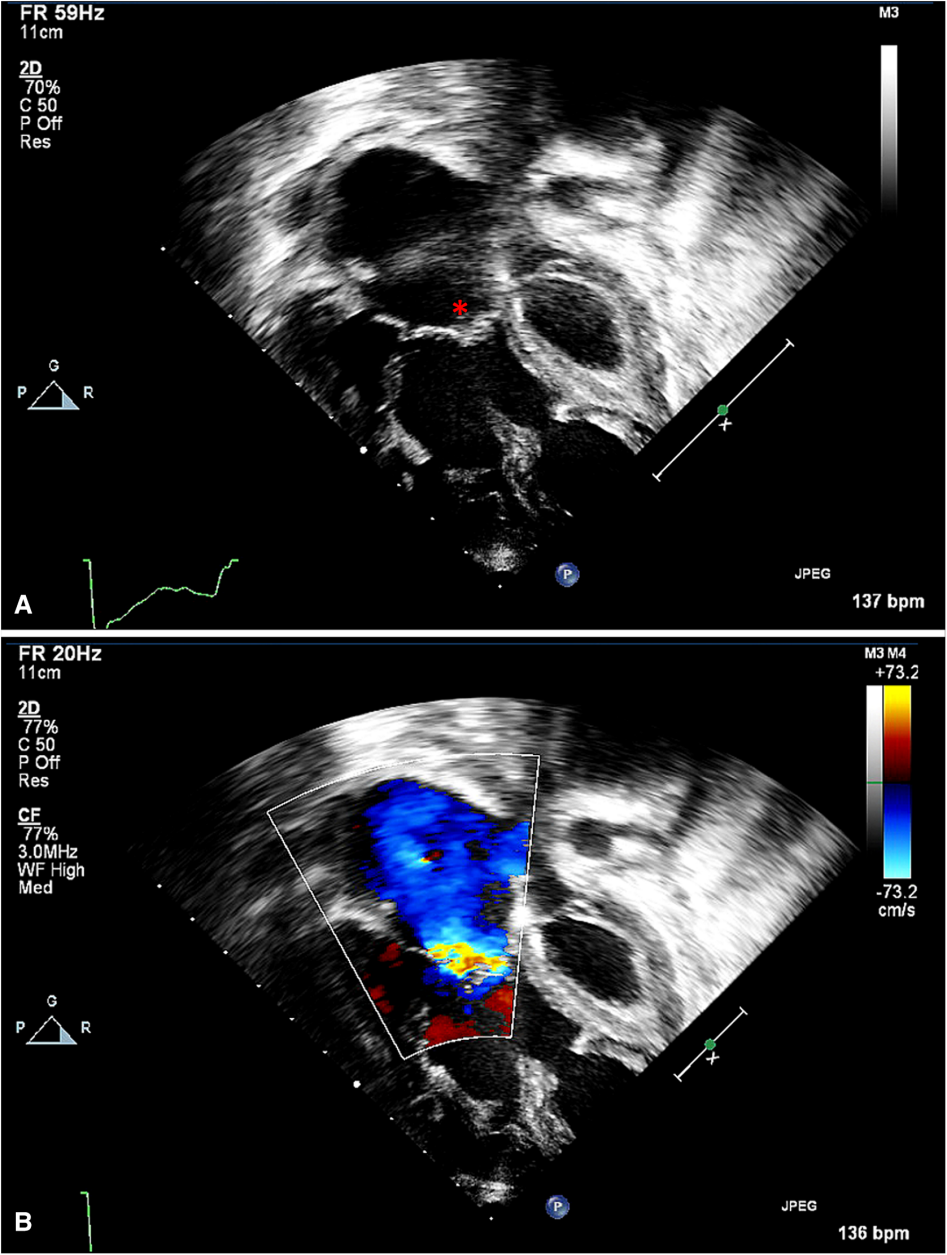
Apical view of tricuspid valve, with annular dilatation. (**A**) The valve's coaptation area is reduced (*). (**B**) The color Doppler interrogation indicates a broader area of regurgitation, through multiple fenestrations in addition to the central area of malcoaptation.

Anatomical factors noted by the echocardiographer which contributed to TR included (Figures 2, 3**)**:

• annular dilatation
• valve prolapse due to either chordal elongation or rupture of a papillary muscle
• leaflet restriction or “tethering”
• valve leaflet dysplasia including presence of clefts in the leaflets
• papillary muscle abnormality
• presence of endocardial fibroelastosis
• right ventricular dysfunction

Ventricular dilatation caused by chronic volume overload and leading to progressive annular dilatation, was also noted.

**Figure 4:**
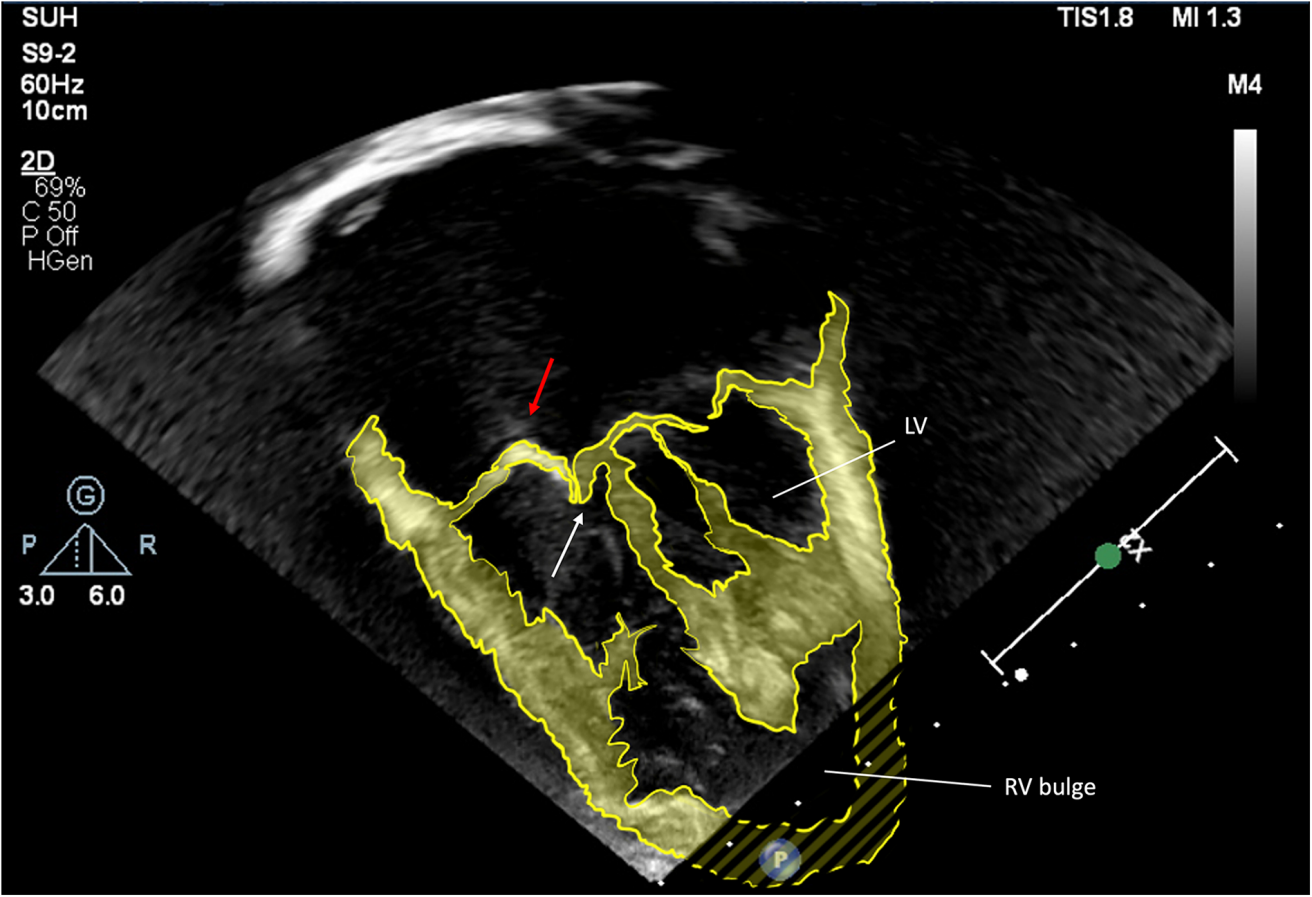
Apical view, enhanced 2D echocardiographic image. Antero-superior leaflet prolapse (red arrow), combined with tethering of the septal leaflet (white arrow). The right ventricular bulge is indicated.

This study showed that echocardiography commonly detects annular dilatation and valve prolapse as primary mechanisms of TR, and also often identifies leaflet dysplasia, but not clefts, and leaflet tethering. During surgery, a different subset of abnormalities, namely annular dilatation and valve dysplasia, were identified as being the most common mechanisms of TR. These findings underline that it is important to obtain detailed echocardiographic studies prior to surgery, in particular to elucidate valve motion abnormalities, which the surgeon does not have the opportunity to view in the flaccid heart attached to cardiopulmonary bypass (Figure 4); however surgical inspection is crucial to complement the echocardiographic information regarding structural malformations, which are often under-recognised on routine imaging.

## Insights from 3D echocardiography

Three-dimensional echocardiography (3DE) provides several insights into TV structure and function that cannot be elucidated by 2D echocardiography alone (20, 21). 3DE demonstrates the complex shape of the TV annulus, which is elliptical and saddle or wave shaped (21, 22). This elliptical shape of the annulus is central to its normal coaptation (23). The septal leaflet has less mobility than the other two leaflets, and therefore if the annular geometry becomes circular, the septal leaflet is pulled away from the other two leaflets, and TV insufficiency is likely to result. The functional elliptical shape is maintained in large part by normal curvature of the septum, which bows towards the RV from the left ventricle. In patients with HLHS, the septal geometry and loading conditions are very different to normal, with resulting loss of the important contribution of geometrical factors to normal competent coaptation.

**Figure 5:**
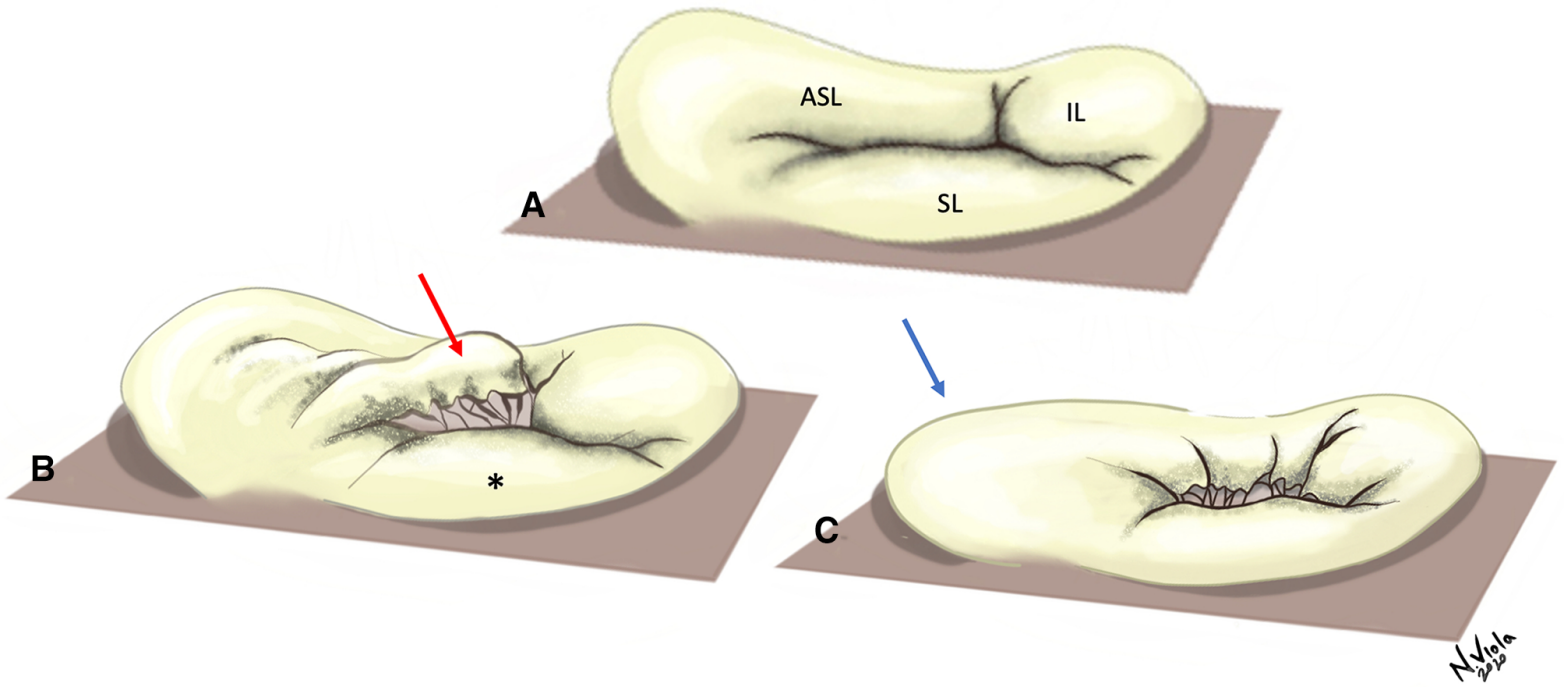
Typical pathology of the tricuspid valve in surgically palliated HLHS. (**A**) Normal TV valve, with a wave-like annulus and normally coapting leaflets. (**B**) Prolapse of the ASL (red arrow) and small SL (*), resulting in inadequate coaptation at the anteroseptal commissure and antero-superior flattening of the annulus. (**C**) Tethering of both SL and ASL, with failure of coaptation and marked flattening of the annulus across the cycle (blue arrow). ASL, Antero-superior leaflet; SL, Septal leaflet; IL, Inferior leaflet.

3DE also has been used to demonstrate considerable evolution of the TV in the “interstage” period between the first and second palliative surgical stages. A very informative study by Takahashi and colleagues (24) used 3DE to investigate the evolution of the TV in patients with HLHS who started with no more than mild regurgitation prior to the first stage of surgical palliation. By the time of the second surgical stage, which is a relatively short period of a few weeks or months after the first palliative stage, they recognised increase in the TV annulus and leaflet area and changes in motion of the leaflets, as well as alteration in the geometry of the subvalvar apparatus. Clinically significant TR of at least moderate degree was associated with tethering and prolapse of the leaflets. Valves with tethering of the leaflets had lateral displacement of the anterior papillary muscle and the TV annulus took on a more planar shape, causing increased pull on the tension apparatus. Prolapse of the leaflets was associated with a smaller septal leaflet area, dilatation of the valve annulus in systole and older age, which is to say it is likely to develop over time (Figure 5).

Early recognition of these abnormalities utilising 3DE has potential to help with prognosis; early leaflet tethering, identified prior to any intervention, is associated with the development of TR (24). Greater degree of tethering of leaflets and annular flattening prior to stage 1 surgical palliation has been shown to be associated with greater degree of TR in medium term follow up, and with reduced survival (7).

These progressive changes in TV features appear to be an adaptive response to the unique loading conditions in the functionally single ventricle circulation. Colen and colleagues used 2DE and 3DE to investigate this adaptation of the TV during the surgical palliation pathway (25). They obtained echocardiographic imaging of HLHS patients prior to their first surgical procedure, the Norwood-Sano operation, at approximately 5 days old, and prior to their second procedure, a bidirectional cavopulmonary anastomosis, at approximately 5 months old. Despite this short time interval, they observed conspicuous changes in the TV, notably increased indexed leaflet and annular area and leaflet tethering volume, but without a resulting change in coaptation. They suggest that the TV in HLHS responds to the haemodynamic stressors it experiences in this interstage period, specifically supranormal preload and afterload, by increasing its leaflet size in order to maintain sufficient leaflet coaptation. However, patients with significant – at least moderate – TR at the time of the second surgical stage were those with greater leaflet size and prolapse, suggesting that an over-exuberant response in leaflet expansion causes this TV maladaptation, and this potentially helpful development becomes detrimental in these patients.

## Ventricular function

Even as early as fetal life, and before and after the first stage of palliation, the RV experiences significant volume loading as compared to a two-ventricle circulation. This significant volume load drives secondary compensatory changes in the RV, including hypertrophy and dilatation (26, 27). The resulting change in RV geometry creates a more spherical configuration, with consequent effects on RV systolic and diastolic function, and TV performance. This more spherical RV has reduced longitudinal deformation compared to circumferential deformation in the fetus, and reduced compliance (28). These indicators of ventricular remodeling are observed also in postnatal life, but it is notable that these changes begin in fetal life, before any opportunity for intervention.

Ugaki and colleagues (19) report a decrease in RV function assessed by RV fractional area change following TV repair, and suggest that this is an unmasking of pre-existing RV dysfunction, exposed by reducing the volume load associated with TR. However, there are other potential factors which could be responsible for the reduction in function at this point in the patient journey, including cardiopulmonary bypass and surgery and perioperative haemodynamic conditions; additionally it is not clear whether this is a temporary or permanent reduction in function. Other studies do not report an important lasting compromise of RV function following TR repair in patients with HLHS (16); this finding may reflect differences in patient selection or post-operative care. It is more clearly evident that the geometry of the RV becomes more favourable with reduction in TR, resulting in smaller RV volume, smaller TV annulus and an improved sphericity index (19). Considerations regarding RV function need to be taken into account in decision-making regarding surgery or timing of surgery for individual patients.

**Figure 6:**
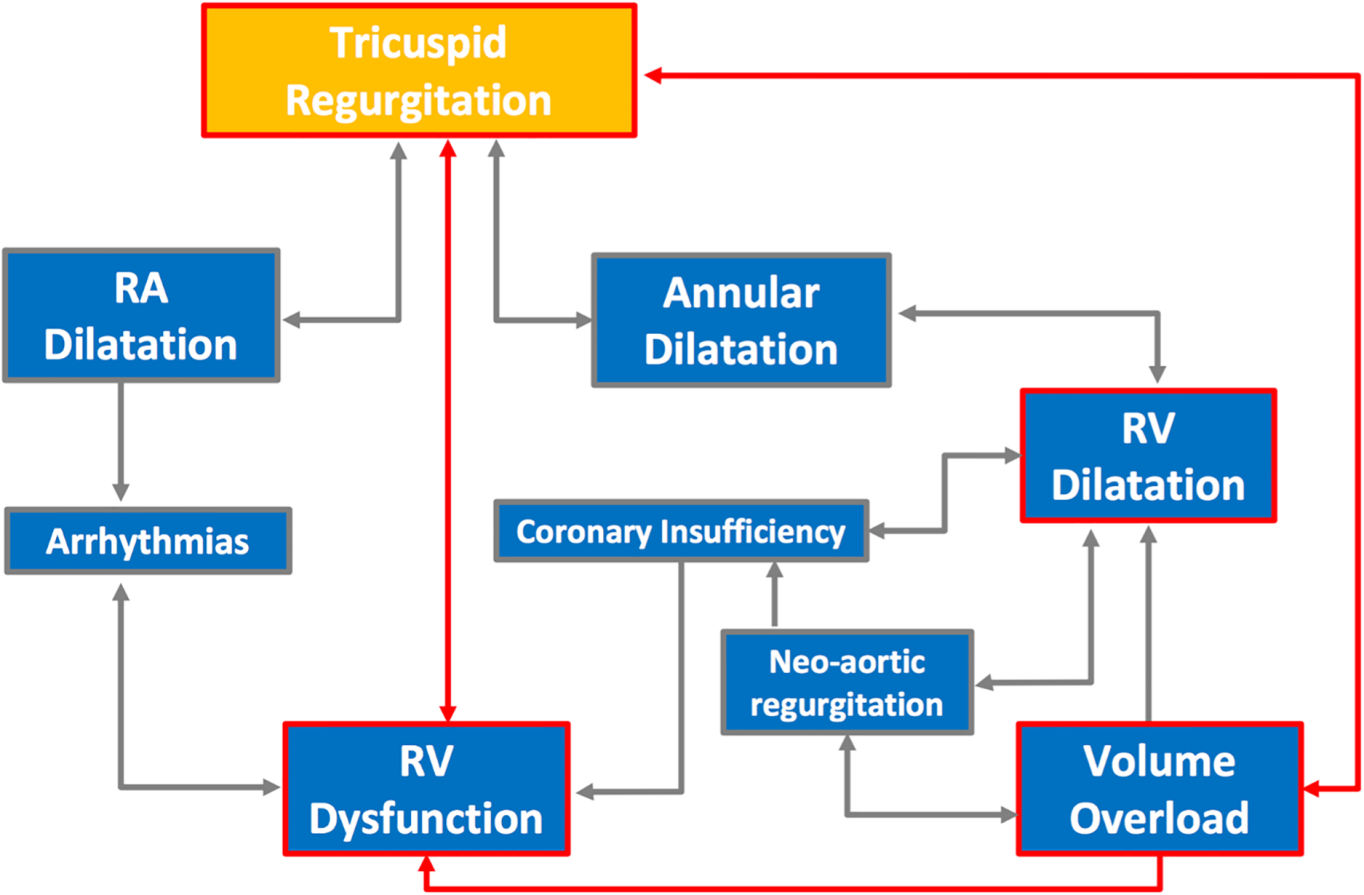
The complex interactions between factors affecting the development of RV dysfunction and its effect on development or exacerbation of TR.

Dysfunction of the RV at the time of the Norwood operation is an independent predictor of poor outcome (29). There is a complex interaction between the function of the RV and the dynamic presence of TR, with multiple interacting factors playing a role in the development or exacerbation of each (Figure 6).

## Advanced echocardiographic imaging

Modern echocardiographic techniques provide additional detail regarding the effects of abnormal contraction patterns and the progressive development of TR. We assessed the presence and degree of asymmetry of contraction between the septal and lateral RV walls using peak strain and difference in time to peak strain (30) in patients with HLHS at various stages of surgical palliation.

Patients with more significant TR had more disparity in the degree of developed strain and delay in time to peak strain around the TV annulus. RV mechanical dyssynchrony and asymmetry in contraction around the TV annulus contribute to changes in the TV shape and motion, and the development or exacerbation of TR. The rate at which changes in ventricular function and severity of TR develop, as monitored on serial echocardiography, is an important marker of poor outcome (31).

**Figure 7:**
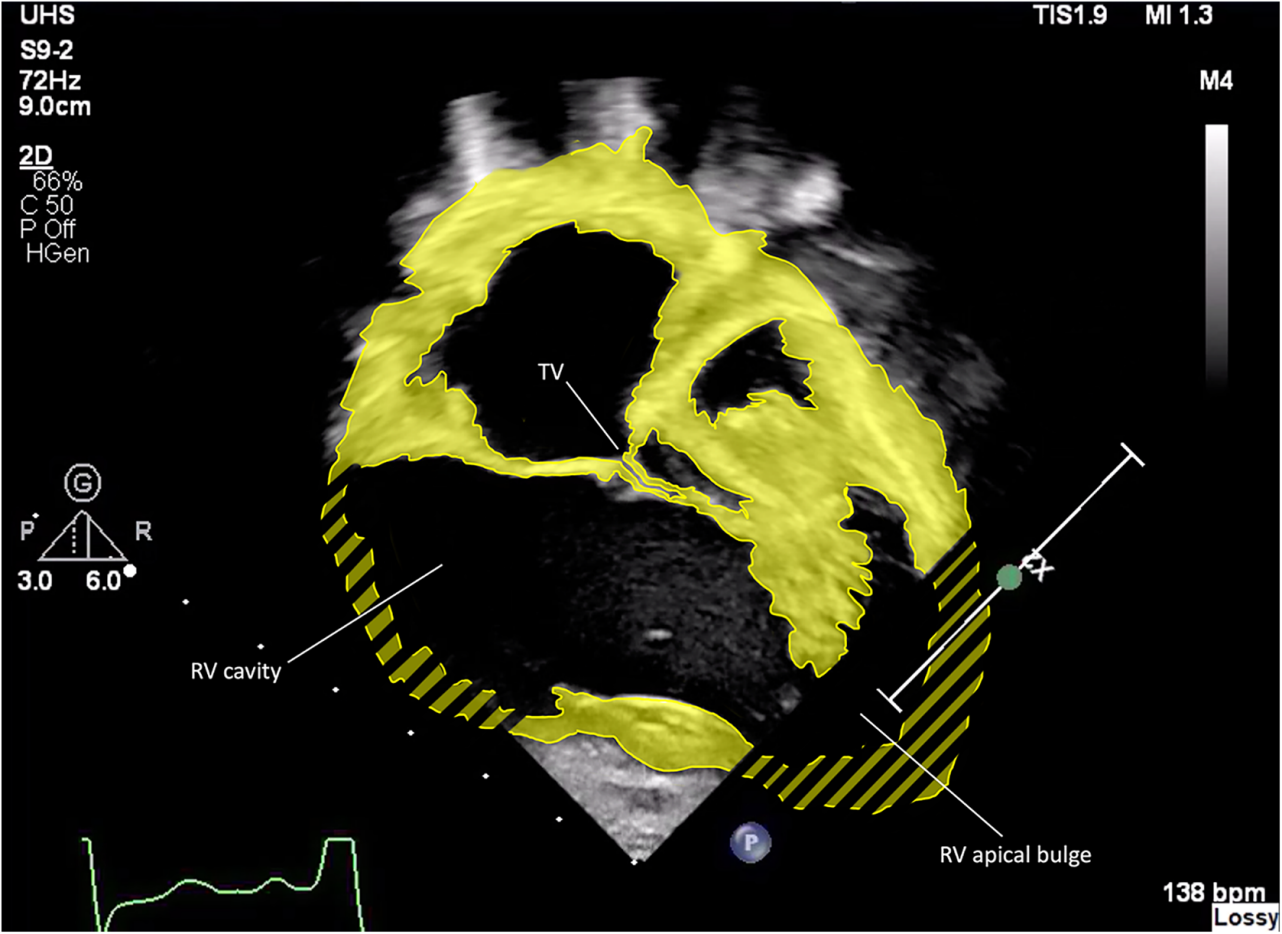
Enhanced 2D echocardiographic subcostal view. Note the RV apical bulge encircling the hypoplastic LV.

Differences are observed between HLHS patients in the geometry of the RV and septum, and in their consequent ability to tolerate or compensate for TR (32). Across all anatomical subtypes, the presence in some patients of a hypertrophied left ventricle creates a sigmoid shaped interventricular septum with “apical bulging” of the RV (Figure 7**)**. We observed in this group a negative effect on regional and global RV function as assessed by RV longitudinal strain, with a pronounced effect on regional dysfunction compared to patients without apical bulging. Severe TR in the apical bulging group was associated with death or transplant, and we hypothesize that ventricles with this geometry may be compromised in their ability to compensate for the additional volume loading that is imposed by the presence of higher degrees of TR.

## Future directions

We have discussed the insights of echocardiography into the evolution of TR in early infancy. The presence of TR remains a risk factor for patients with HLHS as they progress along the single ventricle palliation surgical pathway (5, 8, 12), however data regarding the ongoing adaptation of the TV into adolescence and young adulthood are lacking, and should be the focus of future studies.

In adult cardiac surgery, repair of mitral valves is greatly aided by the use of transoesophageal 3DE (33), and automated or semi-automated segmentation enormously increases the clinical feasibility of its use. Transoesophageal 3DE is limited in small children and infants by lack of suitable 3D probes, and transthoracic 3DE typically produces lower quality imaging with reduced temporal and spatial resolution. However, human expertise paired with evolving machine learning techniques may produce forthcoming advances this area (34). The ability to use transoesophageal 3DE to display anatomy and function of the tricuspid valve and rapidly analyse pathological features amenable to surgery would be extremely beneficial. Transoephageal echocardiography lends itself well to intra-operative use, as general anaesthesia is usually required in children.

3D printing is beneficial in planning of complex congenital cardiac surgical procedures, but mainly relies on data obtained from CT scans. Developments using transoesophageal 3DE as the substrate for 3D printed models allow the creation of bespoke models of congenitally malformed valves, including in patients with HLHS (35). These models allow the surgeon to clearly understand the individual patient's anatomy, and also to plan and simulate the operation, as well as to teach important surgical techniques. These models are currently constrained by limitations in spatial and temporal resolution, availability of software, expense and processing time, but future developments may improve their clinical utility and accessibility.

## Conclusion

TR is common in HLHS at all stages of palliation, and is the result of a combination of anatomical abnormalities of the TV and of the haemodynamic circumstances of its role in the systemic RV in the functionally univentricular heart. Variations in the geometry of the RV and the TV annulus, and the influence of the rudimentary LV, have an important impact on the mechanical function of the TV. The TV adapts and evolves during the course of surgical palliative treatment. 2D and 3D echocardiography, together with newer techniques including strain imaging, provide insight into the morphology of the TV, mechanisms of TR, and the interactions with the unique loading conditions of HLHS. TR has a detrimental effect on patient outcomes and is a potential surgical target, with effective surgical treatment conferring a survival benefit. Recognition of specific mechanisms of TR can direct precise surgical interventions and inform the best timing for intervention on the valve, and therefore enhance patient-specific care.
